# The Emergence of a Universal Rhythmic Feature: Simple Models Can Produce Categorical Rhythms

**DOI:** 10.1111/nyas.70262

**Published:** 2026-04-07

**Authors:** Chloé Coissac, Laura Ferreri, Marco Gamba, Andrea Ravignani, Yannick Jadoul

**Affiliations:** ^1^ Department of Human Neurosciences Sapienza University of Rome Rome Italy; ^2^ PhD program in Behavioral Neuroscience Sapienza University of Rome Rome Italy; ^3^ Department of Brain and Behavioral Sciences University of Pavia Pavia Italy; ^4^ Department of Life Sciences and Systems Biology University of Turin Turin Italy; ^5^ Center for Music in the Brain, Department of Clinical Medicine Aarhus University Aarhus Denmark; ^6^ Research Center of Neuroscience “CRiN‐Daniel Bovet” Sapienza University of Rome Rome Italy; ^7^ Institute of Cognitive Sciences and Technologies National Research Council Rome Italy; ^8^ Artificial Intelligence Lab Vrije Universiteit Brussel Elsene Belgium

**Keywords:** cricket stridulation, insect synchronization, music cognition, neuron model, small‐integer ratios, spiking neural networks, temporal sequences

## Abstract

Across musical cultures, rhythm consists of discrete categories of interval durations. Such rhythmic categories are also increasingly quantified in various nonhuman species’ displays. However, their evolutionary origins are still largely unknown. Complementing cross‐species comparative work with computational modeling can help us understand the cognitive mechanisms underlying the emergence of this universal rhythmic feature and the minimum requirements for producing it. This study investigates whether minimal computational models can produce rhythmic categories. We compare two computational models: a single spiking neuron model representing a minimal neural system, and a model of cricket stridulation as a minimal synchronization mechanism. Both models transform a random temporal sequence into a more structured, isochronous rhythm; that is, randomly distributed temporal intervals get more similar in duration. An isochronous input sequence, in contrast, combined with the models’ intrinsic bias, has a more complex effect on the produced temporal patterns. At frequencies that closely relate to the models’ intrinsic frequency, the models produce stable temporal patterns with rhythmic categories. Our results show that rhythmic categories can emerge from simple mechanisms, likely shared across species, especially when multiple individually isochronous mechanisms interact. As such, we should expect to find categorical rhythms across an even larger range of animal displays.

## Graphical abstract

We investigated the rhythmic structure of temporal sequences generated by two simple models: a spiking neuron model and a model of cricket stridulation. More specifically, we quantified the presence of rhythmic categories in the output sequences of the two models. We studied the models’ rhythmic behavior when given two different kinds of temporal input sequences: random and isochronous input sequences demonstrate the models’ intrinsic biases and emergence of rhythmic categories.

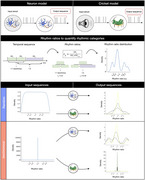

## 1 Introduction

Music pervades all cultures of the world and often plays a significant role in people's lives [1, 2, 3]. Both producing and listening to music require a large set of fine‐tuned, intertwined sensorimotor and cognitive abilities [4, 5, 6]. Yet, as cognitive traits do not fossilize, the origins of musicality, when and why it emerged in our species, still remain largely unclear [7, 8, 9].

To better understand the complexities surrounding the origins of musicality, one practical approach is to deconstruct music—defined as the musical material produced by humans based on their musicality and shaped by various cultural and social constructs [7]—into its components, such as rhythm, melody, and timbre. By examining each component in isolation, researchers can identify whether specific features are common across different musical cultures or unique to a particular one [2, 3, 10]. The features shared across cultures provide a foundation for exploring the origins of music in our species. These universal features may reflect musical traits influenced by general biological or cognitive constraints, indicating mechanisms that existed before the cultural specialization of music [1, 7, 11].

Rhythmic categories are an example of a universal feature of the rhythmic component of music, potentially related to the musicality traits of beat and meter [12, 13]. Across musical cultures, temporal interval durations are not randomly distributed; instead, they are frequently clustered into discrete categories. These categories often conform to small‐integer ratios, with intervals whose relative durations can be expressed as fractions of two small‐integers [2, 14, 15]. For example, in Western music, a half note has double the duration of a quarter note; that is, these are two categories of interval durations conforming to a 2:1 integer ratio, a frequently encountered small‐integer ratio. In other musical cultures, similar rhythmic categories exist, even though the integer ratio relationships between them can vary [2]. In brief, rhythmic categories appear fairly universal in humans and can be considered as a human musical trait.

Rhythmic categories and small‐integer ratios can be investigated through cross‐species comparative studies: if two species share a trait, this trait may have developed under similar circumstances [16, 17]. A trait present in phylogenetically close species may suggest it was present in a common ancestor; the same trait found in more distant species may indicate convergent evolution due to shared ecological constraints [18, 19]. Rhythmic categories aligning with small‐integer ratios have been found in behavioral displays of nonhuman primates [20, 21], other mammals [22], birds [23], and insects [24, 25]. This suggests that rhythmic categories, often following small‐integer ratios, may arise from shared ancestral mechanisms or diverse, coevolved mechanisms, rather than being uniquely human. In both cases, rhythmic categories may occur for two reasons. According to the biological constraints hypothesis, our perceptual and motor systems shape what temporal pattern we can detect and produce. For example, in humans, neural mechanisms [26, 27, 28] and motor systems [29, 30] may favor periodic, integer‐ratio structures, because they are easier to perceive and produce [14]. Alternatively, according to the social coordination hypothesis, rhythmic categories evolved to support synchronization and coordination needs between individuals [31]. Rhythmic categories, especially those based on simple integer ratios, support sustainable alignment in time by providing predictable, shared, and stable temporal structures [32].

Given the growing evidence of rhythmic categories across species, but the lack of explanatory cross‐species mechanisms for this trait, computational models have strong potential to push forward our understanding of the emergence of rhythmic categories. Computational models and experiments enable the detailed exploration of the potential mechanisms that constitute a trait [33, 34, 35]. Furthermore, comparing several models that explain the same trait can refine the mechanistic explanation of a trait and help compare theoretical assumptions [36, 37]. Thus, modeling possible underlying mechanisms of cognitive traits is a crucial step in uncovering the origins of musicality.

Here, we employ computational models and simulations as a complementary approach to cross‐species behavioral studies to understand how rhythmic categories and small‐integer ratios arise. Computational, generative models based on simple hypothesized mechanisms can show whether and how rhythmic behavior emerges from basic principles. The overarching question is: Which mechanisms are necessary and sufficient for producing rhythmic categories and rhythmic patterns conforming to a small‐integer ratio? Previous modeling work followed two complementary strategies.

A first strategy is to start from basic physical principles and incrementally add complexity to the model; for example, as in the neural resonance theory, modeling how the human brain processes and synchronizes to external rhythmic stimuli [28]. This theory posits that rhythmic structure emerges from the dynamics of neural circuits, modeled as systems of oscillators [38, 39]. Over time, these models have been enriched by adding complexity, such as Hebbian plasticity [40], to improve their resemblance to observed behavior. This kind of model aligns with the biological constraints hypothesis regarding the emergence of integer ratio categories. It demonstrates how constraints of the sensorimotor system, rooted in the biophysics of groups of neurons, can give rise to structured rhythmic output, including integer ratios [28, 38, 39]. As such, minimal computational neural models can provide a biologically plausible basis for exploring how rhythmic behaviors emerge from biological constraints, without requiring specific neural architectures, and are applicable at a cross‐species level.

When modeling neurons as a dynamic system of oscillators, one particularly relevant property is mode locking: When one neural oscillator is driven by a periodic input signal (also known as “periodic external forcing”), it will—under certain conditions—produce a stable firing pattern, which relates to the input stimulus at an integer ratio. More precisely, when two oscillators are *n*:*m* mode‐locked, for each *m* cycles of the forcing input, the forced oscillator completes *n* cycles [41]. The different stable zones of mode locking in such a system can be visualized in the system's parameter space and often have an elongated, triangular shape, known as Arnold tongues [42]. This mode‐locking behavior thus corresponds to an integer ratio relationship between the input stimulus frequency and the oscillator's average output frequency.

However, the mode‐locking behavior of neural oscillators does not directly correspond to the presence of small‐integer ratios in the temporal patterns produced by the forced oscillator. As such, the link between the mode‐locking behavior of such models and the previously described rhythmic categories in human music and animal behavioral displays remains unexplored. By quantifying the presence of rhythmic categories and small‐integer ratios in the temporal sequences produced by simple neural oscillator models, we aim to bridge this gap. This approach can connect the behavioral observations of rhythmic categories and the existing knowledge on the dynamics of systems of oscillators and neural resonance theory as a model of the underlying internal mechanisms.

A second possible modeling strategy begins with empirical observations of rhythmic animal behavior and derives a mathematical abstraction of that behavior. For example, the stridulation patterns of a tropical cricket have inspired a model of synchronization in this species (*Mecodopa* species *S* [25]). In this model, the cricket adapts its chirping period to synchronize at an integer ratio with conspecifics. Based on empirically measured responses to artificial stimuli, this behaviorally derived model abstracts the biological behavior and makes the underlying dynamics tractable. The analysis of this cricket model shows that it also exhibits mode‐locking behavior at small‐integer ratios^1^ [25]. This behavioral model in particular relates to the social coordination hypothesis, as it examines the potential role of interindividual synchronization mechanisms in shaping rhythmic patterns. Doing so, we can investigate how basic synchronization mechanisms influence the generation of temporal sequences with rhythmic categories and small‐integer ratios.

In this study, we present and test two computational models, one for each of these two modeling strategies. The first model is a spiking neuron model—henceforth referred to as the “neuron model”—and simulates the basic rhythmic properties of a single neuron, testing whether a minimal neural system can give rise to rhythmic categories. The second model is a generative version of the previously presented model of cricket stridulation [25]—henceforth referred to as the “cricket model”—and specifically allows us to focus on whether and how simple rules of temporal adjustment can produce rhythmic categories.

Crucially, our two implemented models are based on models coming from two distinct theoretical frameworks and are at two different levels of abstraction. The neuron model originates as a more mechanistic approach and models a real biophysical mechanism. The cricket model, on the other hand, is a mathematical abstraction proposed to reproduce the crickets’ synchronizing behavior, composed of a set of equations that do not represent a concrete mechanism inside a cricket's brain. In this sense, the neuron model—or any other type of low‐level mechanistic model—could in principle underlie the behavioral cricket model, and these two models are not mutually exclusive. However, the set of equations composing the cricket model captures basic synchronization rules and the emergence of mode‐locking behavior in an interpretable way, making it an interesting complementary model to the neuron. Additionally, both models are similar enough to each other to be tested under similar conditions, allowing for meaningful comparison of their results. Simple models are particularly important to develop an understanding of rhythmic categories, as their results can be interpreted across species without assuming specific cognitive capacities. While the presented models are not meant to realistically model neural dynamics or specific behavior of a species, they allow us to isolate which simple biophysical or behavioral mechanisms are necessary and sufficient for rhythmic categories and integer ratios to emerge, and help determine whether these rhythmic patterns are the result of specialized adaptations or emerge from more basic, general‐purpose mechanisms.

## 2 Materials and Methods

### 2.1 Model Implementations

#### 2.1.1 Neuron Model

Our neuron model is based on the leaky integrate‐and‐fire (LIF) neuron model with alpha‐shaped input currents^2^ (see Tables S1 and S2 for a detailed overview), as derived from the NEST spiking neural network simulation software (version 3.8) [43]. The LIF family of neural models is widely used because it combines computational simplicity with the general features of a biological neuron [44, 45]. Although this model is one of the simpler ones available, it already offers a wide range of parameters that we can explore in our experiments and simulations, and this type of model is known to exhibit mode‐locking behavior and Arnold tongues [46, 47].

The central aspect of such an LIF neural model is its membrane potential, a value that evolves over time. Once this potential reaches a predefined, fixed threshold, the neuron fires; that is, it sends a spike to the synapse of any other connected neuron, resets its potential to the baseline potential, and waits a fixed refractory period before repeating this process. Increases in membrane potential can have two causes: a fixed continuous input current or the reception of an excitatory input spike from another neuron. Decreases in potential come from leakage (i.e., modeling the imperfect impermeability of the neuron's membrane), at a rate proportional to the current potential value, and from inhibitory input spikes. This combination of factors makes the charging of the membrane potential a nonlinear process. Note that in the absence of incoming spikes, a single neuron with a fixed input current has an isochronous (i.e., consisting of equally spaced temporal events) oscillator‐like behavior. The isochronous firing rate of such a neuron depends on the incoming current, and our LIF neuron is an instance of a Type I neuron model: once the minimum threshold of input current is reached, the neuron will have a relatively low firing rate and continuously increase this rate as the input current increases [41, 48, 49]. Excitatory and inhibitory input spikes will, respectively, advance or delay the neuron's next firing, and modify its isochronous behavior. The influence of these input spikes is moderated by two additional connection strength parameters: the connection weight and synaptic rise time, which determine the total increase in potential and the duration over which the input spike has an effect.

**FIGURE 1:**
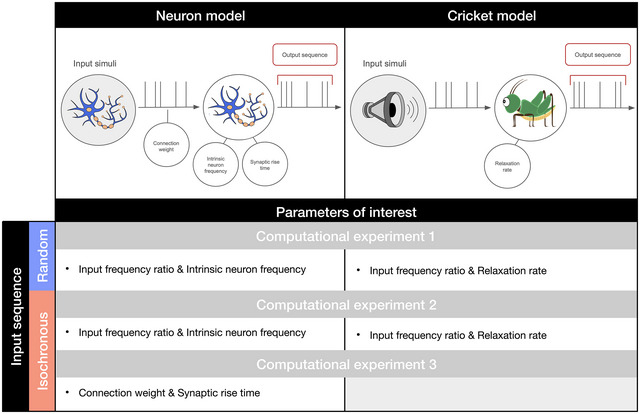
An overview of the two models, the different computational experiments and input sequences, and the parameters of interest manipulated in each experiment (see also Tables **S3−S5** for an overview of the model parameters). Input frequency ratio: In experiment 1, with a random input sequence, the input frequency ratio represents the ratio between the frequency of our models and the average frequency of the Poisson generator. In the context of an isochronous input sequence (i.e., experiments 2 and 3), the input frequency ratio represents the ratio between the model's intrinsic frequency and the frequency of the isochronous input sequence. Following the rhythm ratio formula by Roeske et al. [23], we quantified the ratio as $f_{input}\;/\;\left(f_{input}+f_{model}\right)$ (i.e., an input frequency of three times the intrinsic frequency, 3:1, would have a ratio of 0.75; equivalently, a slower input than the model's frequency results in a ratio below 0.5). Intrinsic neuron frequency: The number of output spikes per second when the neuron does not receive any input spikes depends directly on the input current. The higher the input current, the higher the intrinsic frequency of the neuron, and vice versa (see Table S3). Connection weight: The higher the connection weight, the more the neuron's membrane potential increases when receiving an input spike. Synaptic rise time: The higher the synaptic rise time, the more time it takes for the neuron's membrane potential to rise after receiving an input spike. Relaxation rate: The relaxation rate parameter modulates the rate at which the simulated cricket will return to its intrinsic period after perturbation. A higher relaxation rate corresponds to a stronger return to the natural frequency of the cricket.

In our simulations below, we focused on a single neuron receiving different types of excitatory input spike trains (see Figure 1) and built simulations using the NEST interface for Python. Since an excitatory input spike can only advance the neuron model's phase, it is a Type I model. This stands in contrast to the cricket model we present below, which has a mechanism that can both advance or delay the phase and corresponds to a Type II model. We adjusted the input current to investigate a range of intrinsic isochronous frequencies in this neuron and explored the effects of connection weight and synaptic rise time parameters on the neuron's rhythmic behavior. The spike trains produced by the model constitute the output sequence, which we analyzed for the presence of categorical rhythms and small‐integer ratios (see below).

#### 2.1.2 Cricket Model

Our cricket model is based on the acoustic behavior of a species of crickets. These crickets tend to produce rhythmic signals of chirps that are synchronous or regularly alternating with those of conspecific individuals, with mode locking at specific integer ratios [25]. This behavior was empirically measured in experiments and modeled with a phase response curve (PRC) [25]. The PRC captures the change of cricket's chirping period in response to an external stimulus, depending on the phase offset of that stimulus. The mechanism of the cricket's stridulation achieves synchronization between different individuals as follows: when a stimulus occurs shortly after a chirp, the cricket extends its next chirp period. Conversely, if a stimulus arrives shortly before its next chirp, it shortens its next period. The cricket's PRC, which can either shorten or lengthen its period, would correspond to the PRC of a Type II neuron model. Both types of neuron models (Types I and II) have been studied in depth and demonstrate qualitatively different dynamic behavior, for example, the mode‐locking behavior under external forcing [41, 48]. Whereas the original model by Sismondo [25] consisted of a purely mathematical formulation and derivation, we here implemented this mechanism in a generative model, simulating the production of chirps by the cricket following the original study's description.

To implement this model, we first programmed a function that fitted the graphical representation of the PRC provided by Sismondo [25]. We added a parameter to the function that controls the level of noise in the PRC curve (Figure S1). We then implemented a simulation of the cricket's chirping over time, in response to an external sequence of stimuli (Figure 1; see also Code fragment S1). In our model, if the cricket hears a stimulus, it adjusts its chirping period according to the PRC. If a second stimulus occurs before it can chirp again, it readapts its period based on the new stimulus. Thus, the cricket responds only to the most recent stimulus. The output sequence of this model is the chirps produced over time by the modeled cricket.

Finally, we generalized the model to include a relaxation parameter, which can be adjusted to make the simulated cricket return faster or slower to its intrinsic period when a chirp interval is not perturbed by a stimulus (cfr., “recovery to the free‐run chirping rate,” Ref. [25]). This relaxation rate ranges between 0 and 1; a relaxation rate of 1 corresponds to the cricket returning instantaneously to its natural chirping period after a perturbed period. In contrast, a relaxation rate of 0.2 corresponds to the cricket adapting its perturbed period to be 20% closer to its natural period at each chirp. The way our model is implemented allows for any kind of time series as input stimuli and simulates the cricket's resulting behavior. The two parameters of interest for this model are the frequency of the external stimuli and the relaxation rate of the cricket model, as we explore how these influence the cricket's synchronization.

### 2.2 Computational Simulations

#### 2.2.1 Computational Experiment 1: Random Input Sequences

Using these two models, we built and ran several simulations, organized in three experiments (Figure 1). In the first experiment, we aimed to investigate the rhythmic properties and biases of our individual neuron and cricket models. To do so, we provided both models with an input sequence of randomly distributed temporal events. In particular, we used a Poisson generator to generate input sequences for the neuron and cricket models; that is, excitatory input spikes for the neuron and stimuli for the cricket model. Since the generated Poisson sequences do not contain rhythmic categories (see below) [50], this experiment aimed to provide insight into the models’ internal biases and tendency to induce rhythmic categories from random input sequences. We systematically varied the ratio between the models’ intrinsic frequency and the average frequency of the Poisson generator (Figure 1 and Table S3), ranging from 1:5 to 5:1, to capture the influence of the relative average tempo on the model production. In addition, we modulated the firing rate of the neuron (Table S3) and the relaxation rate of the cricket model.

#### 2.2.2 Computational Experiment 2: Isochronous Input Sequences

In our second experiment, we provided isochronous input sequences as input events to both models in order to study how one isochronously firing neuron or chirping cricket would affect the behavior of another neuron or cricket. Isochronous sequences represent the most structured and least complex form of input, standing in contrast to the random Poisson sequences from the previous experiment. Isochronous sequences are commonly found in everyday biological and physical phenomena (e.g., heartbeats and pendulum oscillations). Our experiment presents a unidirectional interaction between two individual models: that is, one neuron sending input events to another neuron, or one cricket synchronizing its chirping to that of another cricket. For both experiments, we systematically varied the ratio between the models’ intrinsic frequency and the frequency of the input events, as we expected this ratio to play a significant role in the temporal properties of the model's output sequences (Table S4). For example, suppose the ratio between the two frequencies corresponds to an integer ratio. In that case, the input events should have less impact on the naturally isochronous behavior of the models since they already have a natural underlying common frequency. In addition, we modulated the same model‐specific parameters as in the first experiment to assess their influence on the resulting output sequences: the firing rate of the neuron and the relaxation rate of the cricket model.

#### 2.2.3 Computational Experiment 3: Neural Connection Strength Parameters

In the final experiment, we again provided isochronous input sequences to our neuron model, but this time we fixed the input frequency to match the neuron's intrinsic firing rate. Instead, we varied parameters of the connection strength between the two neurons: the connection weight and the output neuron's synaptic rise time, corresponding, respectively, to the size of the increase in membrane potential and to the time over which this increase is spread out (Table S5). These two parameters modulate the connection between the two neurons. Previous analyses have indeed found that such parameters have a strong effect on the temporal pattern produced by a neuron, as a strong and long synaptic excitation can carry over and affect multiple cycles of the receiving neuron [51, 52]. Consequently, we also expected these two parameters to have a substantial impact on the firing patterns and resulting rhythmic categories of the output neuron. The cricket model does not have corresponding parameters; therefore, we focused our attention solely on the neuron model in this third experiment.

### 2.3 Analysis of the Simulations

#### 2.3.1 Analysis of Output Sequences

Both our models produced temporal event sequences. Throughout our analyses, we only considered the output events of each model—the neuron's spike times or the cricket's chirps (Figure 1). As we are interested in the rhythmic categories and potential small‐integer ratios between temporal intervals, we first calculated the interval durations $i_{k}$ between successive events: $i_{k}=t_{k+1}\;-t_{k}$, where $t_{k}$ is the time at which the event k occurs in a temporal sequence. Subsequently, the rhythm ratios $r_{k}$ of a sequence were then calculated using the formula by Roeske et al. [23]: $r_{k}=\frac{i_{k}}{i_{k}+i_{k+1}}$. These values, called rhythm ratios, capture the relative duration of two successive intervals. For example, two intervals with durations following a 2:1 integer ratio would result in a rhythm ratio equal to 0.666…; perfect isochrony, with two subsequent intervals of equal length, corresponds to a rhythm ratio of 0.5. In all our simulations, we generated temporal sequences with at least 100 intervals to provide a sufficiently large sample size.

#### 2.3.2 Analysis of Rhythm Ratios

We used kernel density estimation (KDE) to estimate the probability density function (PDF) of the observed ratios in a nonparametric manner. We calculated it using the *gaussian_kde* function from the SciPy Python package (with the default bandwidth selection). We also calculated the weighted count of on‐integer and off‐integer rhythm ratios (i.e., $r_{k}$ values close to small‐integer ratios vs. those further away from them) for all output sequences, following the approach of Roeske et al. [23]. We did so for the integer ratios 1:3, 1:2, 2:3, 1:1, 3:2, 2:1, and 3:1. However, we did not combine the observation counts of inverse ratios; that is, two ratios such as 1:2 and 2:1 are analyzed separately (following, e.g., Ref. [20]). For each small‐integer ratio of interest, we divided the weighted on‐integer count by the sum of the weighted on‐ and off‐integer counts. The closer to 1 the division is, the more concentrated the PDF on the integer ratio of interest.

Additionally, we also estimated the differential entropy of the observed rhythm ratio distribution in the output sequence. Differential entropy is a generalization of (Shannon) entropy for a continuous probability distribution. Since the rhythm ratios are always between 0 and 1, the maximum reachable different entropy is equal to 0, which corresponds to a uniform distribution achieved by a Poisson process [53]. The more a distribution is organized and thus predictable, the lower its differential entropy, and thus the more negative it is. As such, the input sequences in experiment 1 have rhythm ratios with a high (almost 0) different entropy, and the input rhythm ratios are maximally random from this perspective. Conversely, in experiments 2 and 3, a perfectly isochronous input has an extremely low differential entropy, as all the PDF of the rhythm ratios is concentrated around 0.5. In our analysis, we estimated the differential entropy of our ratio sequence using the *differential_entropy* function in the SciPy Python package.

These measures were calculated to quantitatively explore the previously mentioned parameters of interest in each experiment (Figure 1). To examine the behavior of the two models and visualize the on‐integer ratio fractions and the differential entropy, we computed heatmaps showing these values for all combinations of the two parameters of interest. These heatmaps allowed for the simultaneous examination of the individual effects and the interaction between the parameters.

## 3 Results

### 3.1 Computational Experiment 1

**FIGURE 2:**
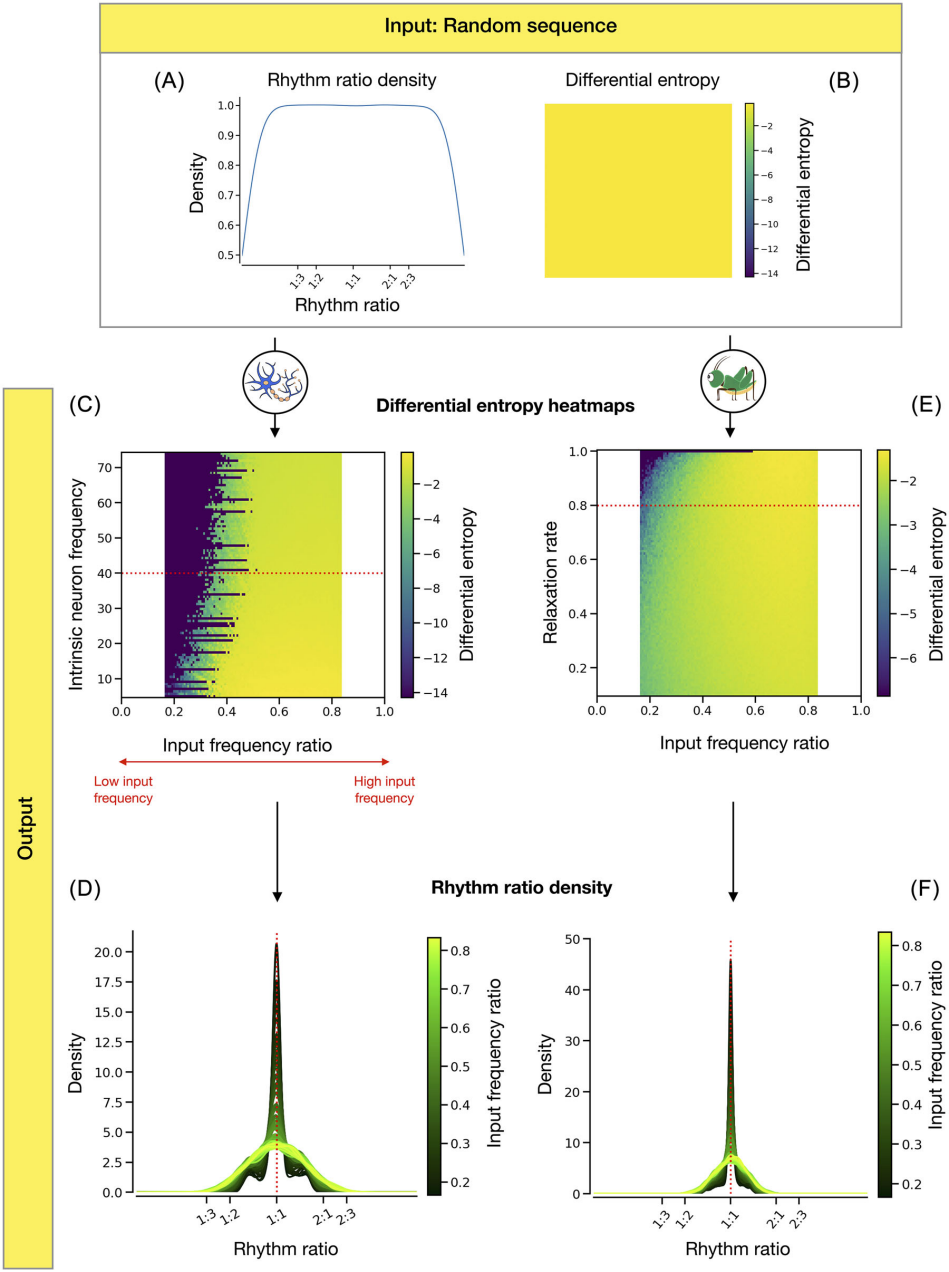
The results of experiment 1 demonstrate how both models have an intrinsic bias toward producing isochrony and reduce the differential entropy of a random input sequence. (A) The estimated probability density of rhythm ratios produced by a Poisson generator is flat, corresponding to a uniform distribution of the ratio between 0 and 1. (B) The corresponding differential entropy of such a random sequence is maximal, corresponding to a yellow color in the heatmaps in panels C and E. (C) The differential entropy heatmap of the output sequences produced by the neuron model shows a decrease in entropy compared to the random input sequences. The color scale is clipped at the minimum finite value, grouping negative infinity entropy estimates together as the same dark purple hue. (D) The estimated probability density functions of the neuron models’ output rhythm ratios highlight the intrinsic bias toward isochrony, increasing as the average input rate decreases. The curves correspond to the simulations in a selected slice of the above heatmap (see the red dotted line on panel B), with a fixed firing rate of the neuron (40) and a varying input frequency ratio. The red dotted line represents the 1:1 ratio (isochrony). (E) The corresponding differential entropy density heatmap for the cricket model broadly shows the same results. Note that the heatmaps in panels C and E are represented on a different color scale, and colors are not directly comparable. (F) The probability density functions of the cricket model support the same conclusions as the neuron model. The density curves represent the slide of the red dotted line in panel E, with a fixed relaxation rate (0.8) and varying input frequency ratio.

In the first experiment, both models were presented with random temporal input sequences to investigate their intrinsic biases. For the neuron model, we varied the relative rate of the input events and the neuron's own frequency. Similarly, we varied the relative input rate and relaxation rate of the cricket model. Across all tested parameter value combinations, the rhythm ratios of both models’ output sequences have notably lower differential entropy (Figure 2C,E) compared to that of their random input sequence (Figure 2A,B). In other words, the output sequences are more organized compared to the maximally random input sequences, indicating that both models have an intrinsic tendency to decrease the level of entropy.

As the average rate of the input sequence increases (i.e., a higher input frequency ratio, *x*‐axis in Figure 2C,E), the organization of the output sequences decreases. If we take a slice of the differential entropy heatmaps (i.e., keeping one parameter's value fixed; see the red dotted lines in Figure 2C,E), we can directly observe the shape of the produced rhythm ratio distributions and see how the models introduce order in the initially random temporal sequences. We fixed the firing rate of the neuron model and the relaxation rate of the cricket model, and varied the input frequency ratio. The resulting rhythm ratio distributions show that both models bias the uniform distribution toward the ratio 0.5 (i.e., 1:1, isochrony) for all the input frequency ratios (Figure 2D,F). This bias toward an isochronous output sequence becomes stronger as the input frequency ratio decreases, and vice versa. Indeed, if the intrinsic frequency of the model is high compared to the average rate of the input stimuli, the effect of the stimuli on the model gradually reduces, and the model reverts to its intrinsic behavior without perturbation: isochrony.

These observations hold across the different values of the fixed parameters (i.e., firing rate and relaxation rate). The heatmaps do not show a strong pattern along the *y*‐axis, suggesting that these two parameters may have a weaker impact on the models’ rhythmic production. Overall, when exposed to a rhythmically random input sequence, both models impose a certain level of temporal structure and produce more isochronous output sequences. Additionally, the lower the input frequency related to the models’ intrinsic frequency, the less impact the input exerts on the models and the more the output will reflect the models’ intrinsic isochronous rhythm.

### 3.2 Computational Experiment 2

**FIGURE 3:**
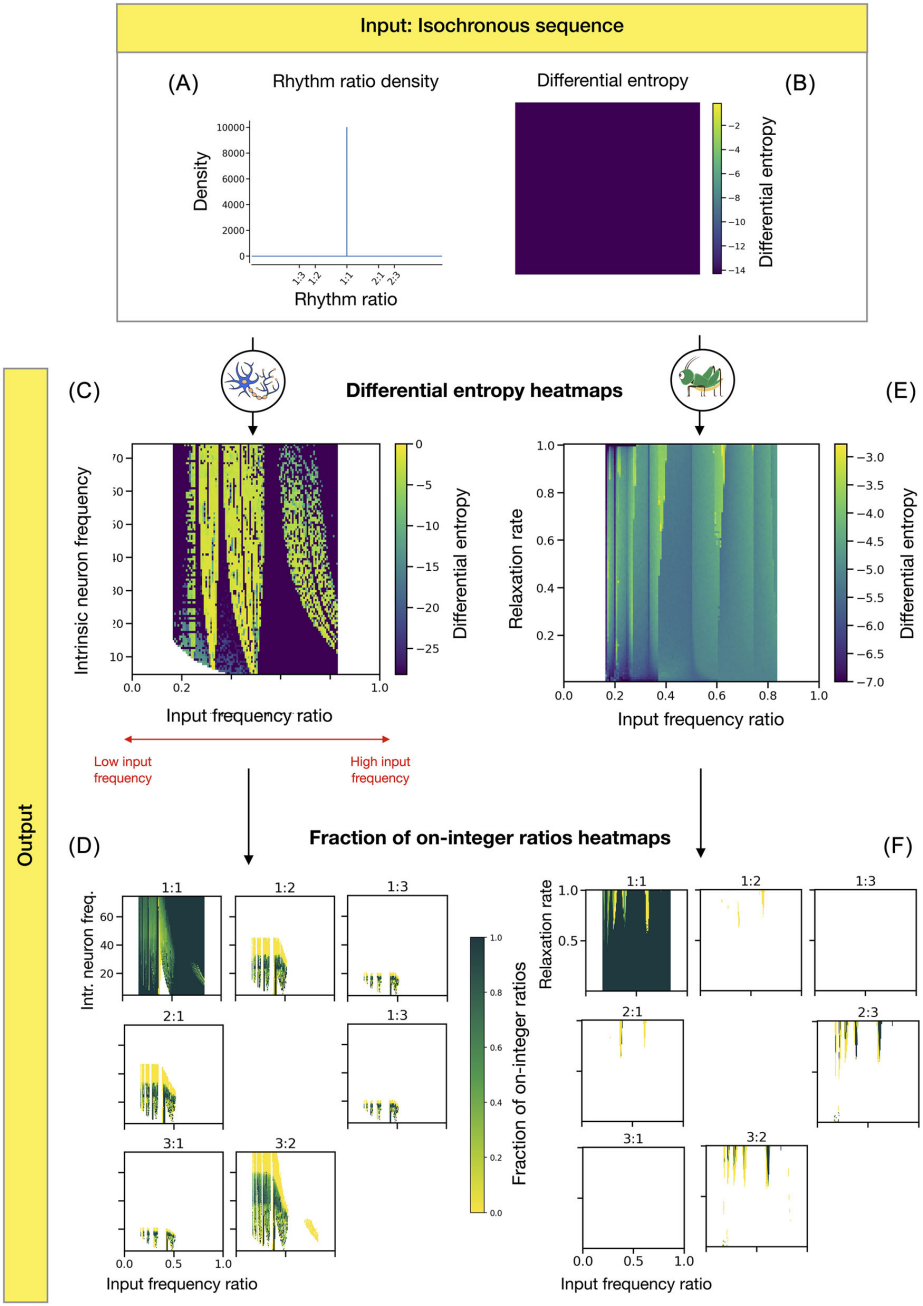
The results of experiment 2 visualize the complex interplay between the two models’ intrinsic frequency and their input frequency, and how this affects the rhythmic structure and generation of small‐integer ratios. (A) The rhythm ratios of a perfectly isochronous sequence are equal to 0.5, and so their probability density function is concentrated around 0.5. (B) The corresponding differential entropy of a perfectly isochronous sequence is minimal (negative infinity), corresponding to dark purple in the heatmaps. For the heatmaps below (panels C and E), the colorscale is clipped at the minimum noninfinite value. (C) The differential entropy heatmap of the neuron model's output shows a complex interaction between the two parameters: regions with highly organized output sequences (in purple) are interspersed with output sequences with a higher differential entropy (in yellow and green). (D) This complex pattern is also visible in the heatmaps displaying the fraction of on‐integer ratios for each of the different small‐integer ratios of interest. For example, the top‐left heatmap shows that a large part of the parameter space is in dark green, indicating that in these regions, almost all of the rhythm ratios around the 1:1 ratio fall close to 0.5 (i.e., fall in the on‐integer bin, following Ref. [23]). (E) The differential entropy heatmap for the cricket model's output overall shows less pronounced differences but still presents a pattern with horizontal strips similar to the neuron model, corresponding to the interaction between the frequency of the model and its isochronous input. Note that the heatmaps in panels C and E are represented on a different color scale, and colors are not directly comparable. (F) For each integer ratio of interest, the heatmaps below present the fraction of on‐integer ratios (analogously to panel D).

In experiment 2, we provided both models with isochronous input sequences. Following our results in experiment 1, isochronous sequences represent the idealized temporal structure each of the two models would produce in isolation. This subsequent experiment thus tested the influence of the input on the model's produced temporal patterns, varying the relative ratio between the input frequency and the models’ intrinsic frequency. Additionally, we also varied the intrinsic neuron frequency and the cricket's relaxation rate. Our results show that the differential entropy of the output sequences varies widely across the parameter space (Figure 3C,E). The perfectly isochronous input sequences correspond to a rhythm ratio distribution concentrated at 0.5 (Figure 3A) and thus have the minimum amount of entropy possible (Figure 3B); yet, in contrast to experiment 1, the models often increase their output entropy due to a mismatch between the input sequence and their intrinsic isochronous rate. A distinct pattern of vertical stripes is visible in both models’ differential entropy heatmap, reflecting this nonlinear influence of the input frequency and the model's intrinsic frequency on the neuron's produced output. The heatmaps reveal zones of total synchronization, where the model and the input fire at the same time and frequency, as well as zones with partial synchronization, where they fire at the same time but with different frequencies at regular intervals.

In the neuron model, regions of low entropy emerge across the heatmap around simple integer ratios (i.e., 1:1, 1:2, 1:3), forming an Arnold tongue‐like structure. In this case, these stable regions occur when the input frequency and the neuron's intrinsic frequency are close to a small‐integer ratio, and the two neurons manage to synchronize, which in turn entails a drop in differential entropy of the output's rhythm ratios (Figure 3C). The tongues are widest for low neuron frequencies and narrow progressively as the intrinsic frequency increases.

**FIGURE 4:**
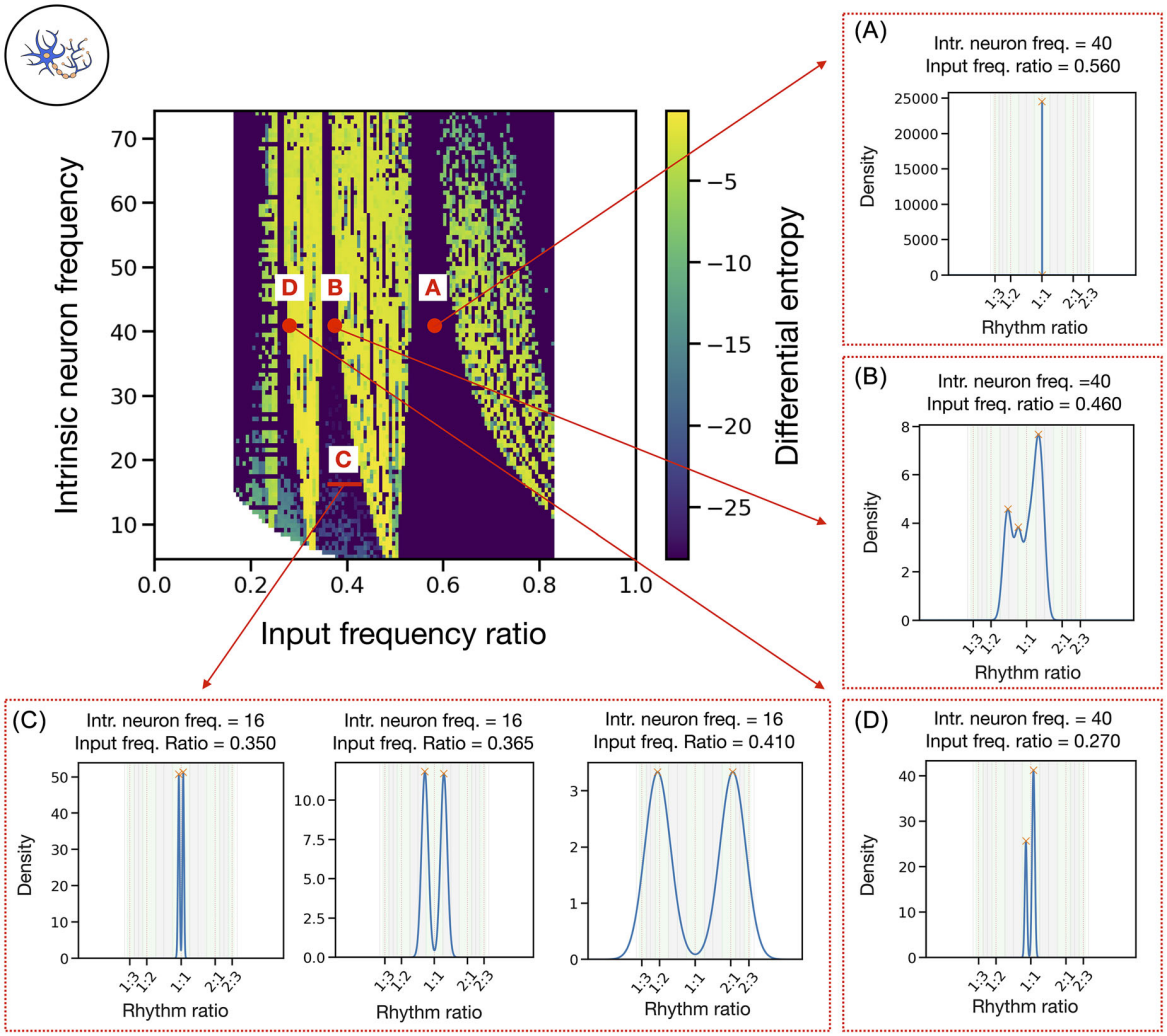
Detailed rhythm ratio density plots highlight the various rhythmic structures produced in different regions of the parameter space for the neuron model. (A−D) Density curves of the rhythm ratios in the neuron model output. Each panel represents a particular region of the parameter space as indicated with red dots or lines in the main differential entropy heatmap (Figure 3C). The background shading behind each KDE curve represents the on/off‐integer bins [23].

In the low‐entropy parameter space region near 1:1 (around ratio 0.5 and higher), we observe a high fraction of on‐integer values for isochrony (represented by dark green, indicating almost all rhythm ratios falling close to 0.5 or 1:1; Figures 3D and 4A). This region corresponds to parameter combinations where the neuron fully synchronizes to the isochronous input. For input frequency ratios slightly above the 1:1 ratios, the input manages to influence the neuron's firing rate by anticipating its activity, gradually pulling it toward its own pace. In the 1:2 tongue (ratio 0.33…), the neuron output is not perfectly isochronous. Two separate clusters of ratios are formed, growing wider and more distant from 1:1 as the ratio between the two frequencies increases (Figure 4C). The neuron is not consistently entrained to the input but instead alternates between two different interval durations. In the region of the parameter space near 1:3 (ratio 0.25), the neuron's output exhibits two asymmetric clusters near the boundaries of the isochrony bin. These measured *r_k_* values correspond to a rhythm consisting of a repetition of three distinct intervals: long, medium, and short (see Figure 4D). Outside of these tongues, the model's output is less organized and less isochronous (Figures 3C,D and 4B). These regions reflect output sequences that seem to lack clear regularity.

The differential entropy of the cricket model does vary systematically across the tested parameter space, but with less marked contrast than we observe for the neuron model (Figure 3E; for a detailed description, see Figure S2 and Results S1). The differential entropy is at its lowest when the frequencies of the model and the input form an exact small‐integer ratio, but we do not observe the same clear Arnold tongue‐like structures as in the neuron model. In between exact small‐integer ratios, the cricket model alternates between several interval durations and produces temporal sequences that are not isochronous but remain much closer to isochrony than the neuron model does (Figure 3F).

In summary, for isochronous input sequences, the rhythmic structure of both models’ output is mainly shaped by the ratio between the stimulus frequency and the models’ intrinsic frequency (i.e., the input frequency ratio parameter, *x*‐axis in Figure 3). The closer the input frequency gets to a model's intrinsic frequency or a small‐integer ratio of that frequency, the more structured the models’ output. In between these ratios, the models introduce some irregularities into the isochronous input. However, the second parameter—that is, the neuron's firing rate or the cricket's relaxation rate, *y*‐axis in Figure 3—also influences the model's output. For the neuron model, lowering the intrinsic frequency broadens the range of input frequency ratios that produce stable output. For the cricket model, lower relaxation rates lead to more stable and isochronous behavior, allowing it to resist perturbations and maintain regular output. Finally, compared to experiment 1, this experiment's results are more complex: Whereas the models mainly demonstrated a bias toward producing isochrony when presented with random input, experiment 2 shows the complex influence of an isochronous input sequence on the isochronous sequences that both models would produce when not perturbed, which can lead to the emergence of distinct rhythmic categories in both models.

### 3.3 Computational Experiment 3

**FIGURE 5:**
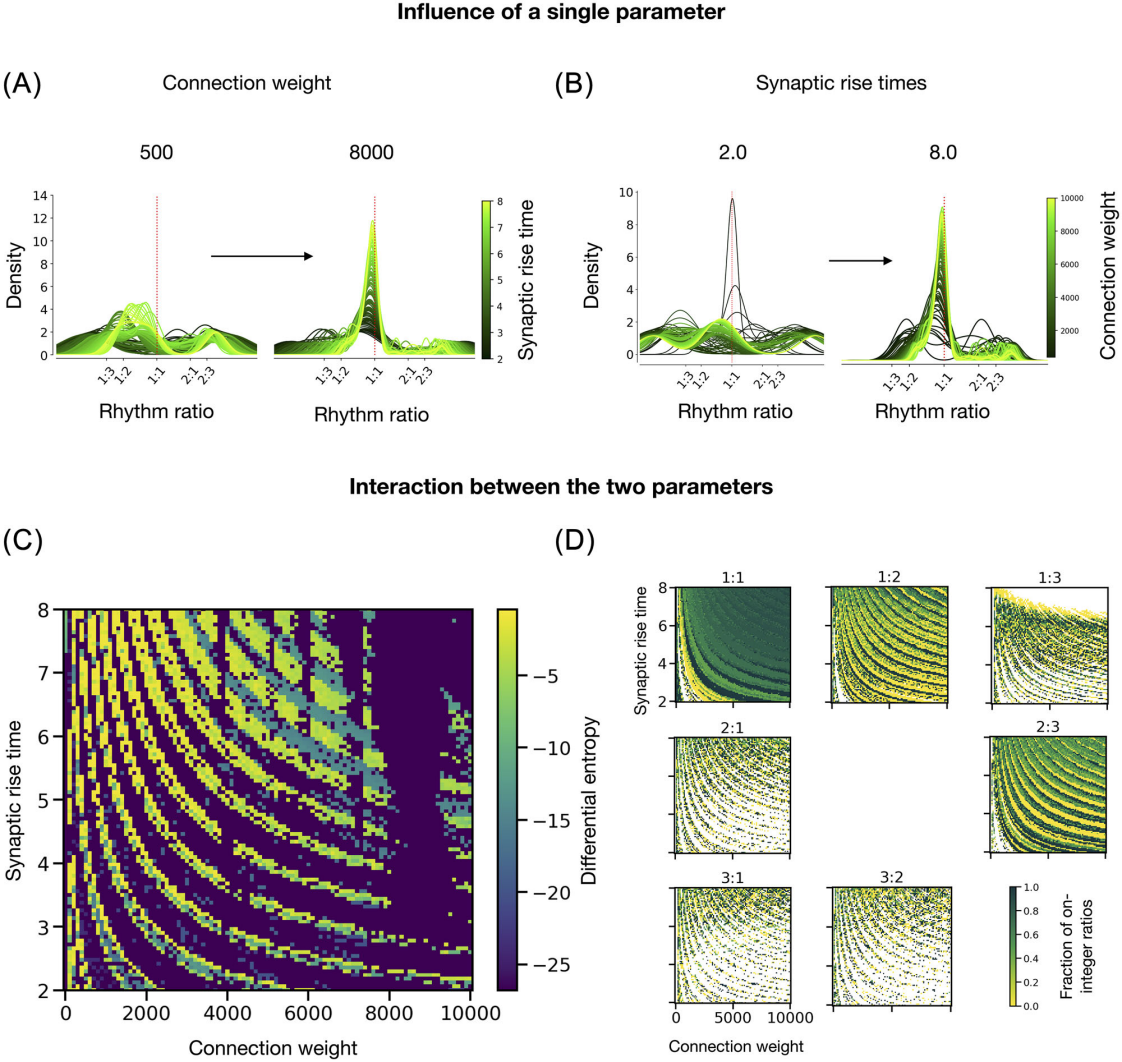
The results of experiment 3 indicate that the output of the neuron model is also strongly influenced by parameters not directly related to the model's intrinsic frequency or input frequency. (A) The densities (KDE) of the rhythm ratios, for two fixed connection weights (500, 8000) and varying synaptic rise times (from 2 to 8 ms). The vertical red dotted lines represent the 1:1 ratio (isochrony). (B) The rhythm ratio densities, for two fixed synaptic rise times (2 and 8 ms) and varying connection weight between the two neurons (from 2000 to 10,000). (C) The differential entropy heatmap of the rhythm ratios from the output sequence for all combinations of parameter values of connection weight (*x*‐axis) and synaptic time rise (*y*‐axis). (D) Analogous heatmaps of the fraction of on‐integer ratios for each of seven selected small‐integer ratios (left to right, top to bottom; 1:1, 1:2, 1:3, 2:1, 2:3, 3:1, 3:2).

In the final experiment, we tested how the connection strength affected the synchronization between the neuron model and its isochronous input. More specifically, we varied the connection weight between the two neurons and synaptic rise time, while providing an isochronous input sequence with the same frequency as the neuron's intrinsic frequency. Comparing the resulting rhythm ratio distributions for two different connection weights (500 and 8000; see Figure 5A), the neuron's rhythm ratio density increasingly clusters around isochrony for the higher connection weight. Indeed, stronger connections enhance the influence of the input neuron and force the output neuron to adjust to its rhythm to match the input frequency, independently of the synaptic rising time.

Similarly, we compare the rhythm ratio densities for two different synaptic rise times (2.0 and 8.0 ms; see Figure 5B). As the synaptic rise time increases, the output neuron's rhythm ratios increasingly cluster around isochrony for any given connection weight. Additionally, when considering the combination of the two parameters in the differential entropy heatmap and the heatmaps with the fraction of on‐integer rhythm ratios, we find a strong interaction pattern (see Figure 5C,D). The interplay between these two parameters creates a complex, striped pattern within the parameter space, characterized by discrete ridges that alternate between high and low differential entropy and on‐integer ratio fractions.

Taken together, these results demonstrate that the two connection strength parameters play an important role in the rhythmic output sequences generated by the neuron model, and that these parameters can play as much of a role as the more obvious frequency‐related parameters. Moreover, these results highlight that the neuron model is not only able to produce isochrony (1:1), but that it can also produce other small‐integer ratios (e.g., 1:2, 1:3, 2:3), even for an isochronous input sequence at the exact same frequency. This can be understood based on the previously referenced studies [51, 52], as higher values for the connection weight and synaptic rise time spread the influence of an input spike across several charging cycles of the receiving neuron. Taken together, these results suggest that the various other parameters of the neuron model, besides the intrinsic frequency, play a decisive yet complex role in creating temporal patterns, and that the model can selectively amplify or suppress certain rhythmic structures based solely on an internal parameter value.

## 4 Discussion

Our simulations tested whether and how rhythmic categories and small‐integer ratios—found across musical traditions and nonhuman animal displays—can emerge from simple, biologically inspired systems. We built two simple models: a model with a single LIF neuron [43] and a generative model of cricket synchronization [25]. We found that both models systematically exhibit a clear bias toward the production of rhythmic categories, even in the presence of a completely random input.

Both the neuron and cricket models’ behavior shows clear similarities as both models bias the ratio distribution toward isochrony (i.e., a 1:1 ratio) when exposed to a random input sequence. This bias is weaker when the mean input frequency was higher than the models’ intrinsic frequency. Indeed, for high enough input frequencies, random input could more effectively perturb the intrinsic rhythm of the models. Interestingly, previous work has found similar effects in human cognition: A study on the effect of auditory distractors (i.e., music) on cognitive performance has found that faster disturbing stimuli interfere more significantly with cognitive processes [54]. Their suggested explanation mirrors our observations, namely, that slower tempi compared to fast ones may be easier to recover from after perturbation or interference.

In contrast, when exposed to an isochronous input sequence—modeling the adaptive behavior to another individual—the behavior of both models is more strongly influenced by the ratio between their intrinsic frequency and that of the input events than by their intrinsic bias toward isochrony. When the input frequency ratio is at or near a small‐integer ratio (i.e., 1:1, 1:2, 1:3), both models reach their lowest output entropy values. These low‐entropy regions reflect zones of increased regularity in the models’ output. These regularities correspond to the creation of similar categorical rhythmic structures for both models: the output sequences feature isochrony, alternating patterns (e.g., short–long, near 1:2 input frequency ratio), or even longer rhythmic patterns (e.g., short–long–very long, near the 1:3 ratio). Isochrony emerges near the 1:1 ratio or when the two frequencies conform exactly to a small‐integer ratio. The two other patterns emerge for input ratios slightly higher than the small‐integer ratios. The results of these simple models align with previous studies on rhythm production in humans. Humans reproduce rhythm better when they are related to integer ratio categories and tend to distort them toward simple ratios if their relations are more complex [14, 55, 56]. In addition, humans are less accurate and precise when synchronizing to complex rhythms that do not conform to simple integer ratios than for rhythms with small‐integer ratios [57].

However, the two models also present some clear differences in their output, both when receiving random and isochronous input sequences. The neuron exhibits a broader range of entropy values and clearly defined areas of low and high entropy. In contrast, the cricket exhibits a lower variation in entropy and a smoother transition between near and far integer ratio regions. This suggests that the cricket model can maintain a more stable output across a wider range of conditions. Another difference lies in the type of rhythmic categories that the models produce. The neuron can produce a wide range of different rhythmic categories, including nonisochronous small‐integer ratio categories also observed in humans and other animals [3, 20]. In contrast, the rhythmic patterns produced by the cricket do not align with small‐integer ratios, but rather show two narrow clusters near isochrony. This corresponds to the model approximately maintaining isochrony, with slight variability across intervals. Similar quasi‐isochronous patterns have been observed in both human synchronization tasks [29] and nonhuman animal vocalizations [24, 58]. One important takeaway message here is that rhythmic categories are not necessarily tied to small‐integer ratios. Rather, some rhythmic categories close to isochrony may emerge from simple strategies aimed at maintaining approximate isochrony.

These differences between the models’ output may—at least in part—be explained by two important intrinsic differences between their mechanisms. The first difference is that the relaxation rate of our cricket model acts as a simple memory component, while our neuron model does not have an analogous mechanism. The neuron model thus quickly returns to its default rate after each perturbation. Meanwhile, at low relaxation rates, the cricket retains the adapted chirping period across several cycles, and only slowly reverts to its natural period. The cricket model also has a Type II PRC, which both advances and delays its phase, depending on the input stimuli's relative phase. These two factors may enable the cricket model to have a smoother transition across ranges of input frequencies and constrain clustering around isochrony, contrary to the neuron model. The second main difference between the two models is that the neuron model presents many more parameters that nonlinearly interact with one another, resulting in a greater range of rhythmic patterns and more abrupt changes in entropy. For example, the two connection strength parameters in experiment 3 resulted in a complex and broad range of varying rhythmic categories across the parameter space. To be able to disentangle the contribution of these intrinsic differences on the temporal production of our models, future work should do a more direct comparison of rhythm ratio production in Type I versus Type II neuron models (see, e.g., Ref. [41]).

Overall, many of the two models’ commonalities can be explained from the perspective of one‐way driven (or forced) nonlinear oscillators [42]. Our two models originate from different approaches: the neuron model is grounded in physical principles, roughly approximating a neuron's processes as an electrical capacitor [59], while the cricket model stems from the empirically observed behavior of the insects. However, their resulting behavior coincides with that of a one‐way driven nonlinear oscillator: When the input frequency is close to an integer ratio reaction with the model's natural frequency, the two systems enter into a stable phase relationship called mode‐locking, creating diverse types of repetitive rhythmic patterns. When the difference between the two frequencies is too far from an integer, the system can no longer maintain a stable behavior; it becomes more variable in its production, entropy increases, and the rhythmic categories tend to disappear. Consequently, we can observe several parallels between the results of our computational experiments and existing theoretical results on neuron oscillator models and mode‐locking behavior in dynamic systems. For example, the entropy heatmaps of the models’ parameter space show structures closely resembling the commonly seen Arnold tongues of mode‐locking forced oscillators [42], and the observed effect of the connection strength between neurons matches previous analogous analytical findings [51, 52]. One important takeaway message is, therefore, that the rhythmic categories observed in our two simple models seem to share several common features with the attractors and mode‐locking behavior of more formally grounded oscillator models [42]. As such, future work should more directly test whether the occurrence of small‐integer rhythm ratios fully coincides with the mode‐locking behavior of these formal nonlinear oscillatory models, or whether these two behaviors can, to some degree, occur independently of each other.

At a first glance, the results of our generative cricket model do not perfectly match the ones presented by Sismondo [25]. Most strikingly, while we do find broad emergence of isochrony and rhythmic categories in the rhythm ratio distribution, our cricket model hardly produces any small‐integer ratios, other than 1:1. However, this discrepancy between the two closely related cricket models can be explained by the way in which the presence of small‐integer ratios is measured. The original study measured the count of chirps or the relative size of intervals between the cricket and the stimulus and found several small‐integer ratios; that is, using dynamic systems terminology, the original study found mode‐locking behavior in the cricket model. In contrast, we investigated the production of small‐integer ratios within the output sequence of the cricket itself (i.e., following previous empirical approaches [20, 23]). These seemingly conflicting results highlight the importance of how to measure small‐integer ratios and which intervals to compare when looking for their presence in various human and nonhuman behavior. When focusing solely on the rhythmic categories within one individual, rhythmic patterns at an interindividual level are easily overlooked. In cases where the goal is to synchronize or communicate with other individuals, future work—both empirical and modeling—should compare the emergence of individual and interindividual rhythmic categories.

Our results do not provide clear evidence against either of the two presented hypotheses on the emergence of rhythmic categories (i.e., the biological constraint hypothesis and the social coordination hypothesis), as both models produce rhythmic categories across a broad range of parameter values. In retrospect, even if our neuron model more closely connects to the biological constraint hypothesis, we observe the most complex rhythmic structure in the simulations where the neuron interacts with structured input from another neuron. Therefore, coordination seems to be an equally relevant aspect for the emergence of rhythmic categories in the neuron model. As such, the model indicates that the two hypotheses are not mutually exclusive; instead, they may offer complementary insights into how both biological and social pressures to coordinate may have shaped the emergence of rhythmic structure. The emergence of rhythmic categories, either at a biological level or a social level, may both be constrained by shared basic coordination dynamics applicable across these two levels [59, 60].

Our simple models produce rhythmic categories, and in some cases, even categories following small‐integer ratios emerge from their simple mechanisms. While such categories are widespread in human musical cultures and also found in a broad range of other animal species [3, 20, 21, 23], their presence in nonhuman animal species is still considered the exception rather than the rule. Given the simplicity of the mechanisms that appear sufficient to produce rhythmic categories, it is surprising that they have not yet been identified in more species’ behavior and displays. This paradox has also been noticed for other rhythmic features such as entrainment and beat‐matching; the ability to match movement to external rhythmic stimuli has so far only been shown in a limited number of species [61]. Similar to our case of rhythmic categories, this lack of entrainment capabilities is unexpected given how widespread mutual entrainment of oscillators is across various biological and physical systems [62]. In general, the mechanisms underlying a rhythmic trait might be simple, but the behavioral expression might depend on additional factors such as motivation, attention, and species–specific auditory–motor constraints [61]. Therefore, our computational models with simple mechanisms show potential minimal requirements for the production of rhythmic categories, but need to be interpreted within the broader context of each animal's specific cognition.

While our models demonstrate that simple neural or behaviorally inspired systems can generate rhythmic categories, they also come with limitations. The cricket model was extended generatively from empirical data; however, its response to several incoming stimuli could be subject to alternative implementations, leading to different behaviors. The neuron model has been chosen for its simplicity, but still contains an extensive range of parameters to explore. A more comprehensive exploration of this parameter space would be necessary. Another limitation is that our selected models exhibit intrinsic isochronous behavior, meaning they already act as oscillators in the absence of perturbing input. This makes it less surprising that the influence of the isochronous input on the models’ behaviors follows the predictions of oscillator theory and that they produce isochronous behavior in response to random rhythm input. Future work should explore whether similar rhythmic categories also emerge in systems that do not rely on intrinsic periodicity or oscillatory dynamics.

## 5 Conclusion

Our study provides computational evidence that rhythmic categories, including those corresponding to integer‐ratio categories, can emerge from simple models based on neural principles and behavioral observations. Our findings show that such categories do not require specific cognition but may arise naturally from general synchronization mechanisms related to interindividual coordination and neural dynamics. Over time, these basic systems may have been reused and refined to support more complex rhythmic behaviors, such as those found in music.

## Author Contributions

Conceptualization: C.C., A.R., and Y.J.; methodology: C.C., A.R., and Y.J; formal analysis: C.C. and Y.J.; investigation: C.C. and Y.J; software: C.C.; writing – original draft preparation: C.C., A.R., and Y.J.; writing – review and editing: C.C., L.F., M.G., A.R., and Y.J.; visualization: C.C.; supervision: L.F., A.R., and Y.J.; funding acquisition: A.R. All authors have read and agreed to the published version of the manuscript.

## Conflicts of Interest

The authors declare no conflicts of interest.

## Supporting information




**Supplementary Materials**: nyas70262‐sup‐0001‐SuppMat.pdf

## Footnotes

1 Note that Sismondo [25] refers to the cricket model's behavior as phase locking. Here, we follow the previously introduced mode‐locking terminology by Farokhniaee and Large [41], with phase locking defined as 1:1 mode locking.

2
https://nest‐simulator.readthedocs.io/en/v3.8/models/iaf_psc_alpha.html
