# Supplementary material for: The Emergence of a Universal Rhythmic Feature: Simple Models Can Produce Categorical Rhythms
Source: Ann N Y Acad Sci. 2026 Apr 7;1558(1):e70262. doi: 10.1111/nyas.70262 (PMC13056270; doi:10.1111/nyas.70262)
Supplement: Supplementary file 1 — Supplementary Materials: nyas70262‐sup‐0001‐SuppMat.pdf [file NYAS-1558-0-s001.pdf]

The emergence of a universal rhythmic feature: Simple models can produce categorical rhythms – Supplementary material

Chloé Coissac[1,2*], Laura Ferreri[3], Marco Gamba[4], Andrea Ravignani[1,5,6,7], Yannick Jadoul[1,8*]

[1] Department of Human Neurosciences, Sapienza University of Rome, Piazzale Aldo Moro, 5, 00185 Roma RM, Italy
[2] PhD program in Behavioral Neuroscience, Sapienza University of Rome, Italy
[3] Department of Brain and Behavioral Sciences, University of Pavia, Pavia, Italy
[4] Department of Life Sciences and Systems Biology, University of Turin, Turin, Italy
[5] Center for Music in the Brain, Department of Clinical Medicine, Aarhus University, Aarhus, Denmark
[6] Research Center of Neuroscience "CRiN-Daniel Bovet", Sapienza University of Rome, Rome, Italy
[7] Institute of Cognitive Sciences and Technologies, National Research Council, Rome, Italy
[8] Artificial Intelligence Lab, Vrije Universiteit Brussel, Pleinlaan 2, 1050 Elsene, Belgium
* Corresponding authors: chloemarie.coissac@uniroma1.it, yannick.jadoul@uniroma1.it

Figure S1: The phase response curve (PRC) of the cricket model, based on the figures by Sismondo.[1]

A: Random variation in the crickets behavior is modeled by the addition of a normally distributed random value when using phase response curve in the model (σ parameter; see Python code in Code fragment S1 below). **B:** The magnitude of the noise added in the PRC affects the differential entropy of the output sequences. Here, three different noise levels and the corresponding differential entropy heatmaps are shown, analogous to the heatmap in Figure 3E. Following experiment 2, the heatmaps show the differential entropy for all combinations of the input frequency ratio and relaxation rate parameters of the cricket model, when given an isochronous input sequence. The level of noise used in our experiments is $\sigma = 0.003$.

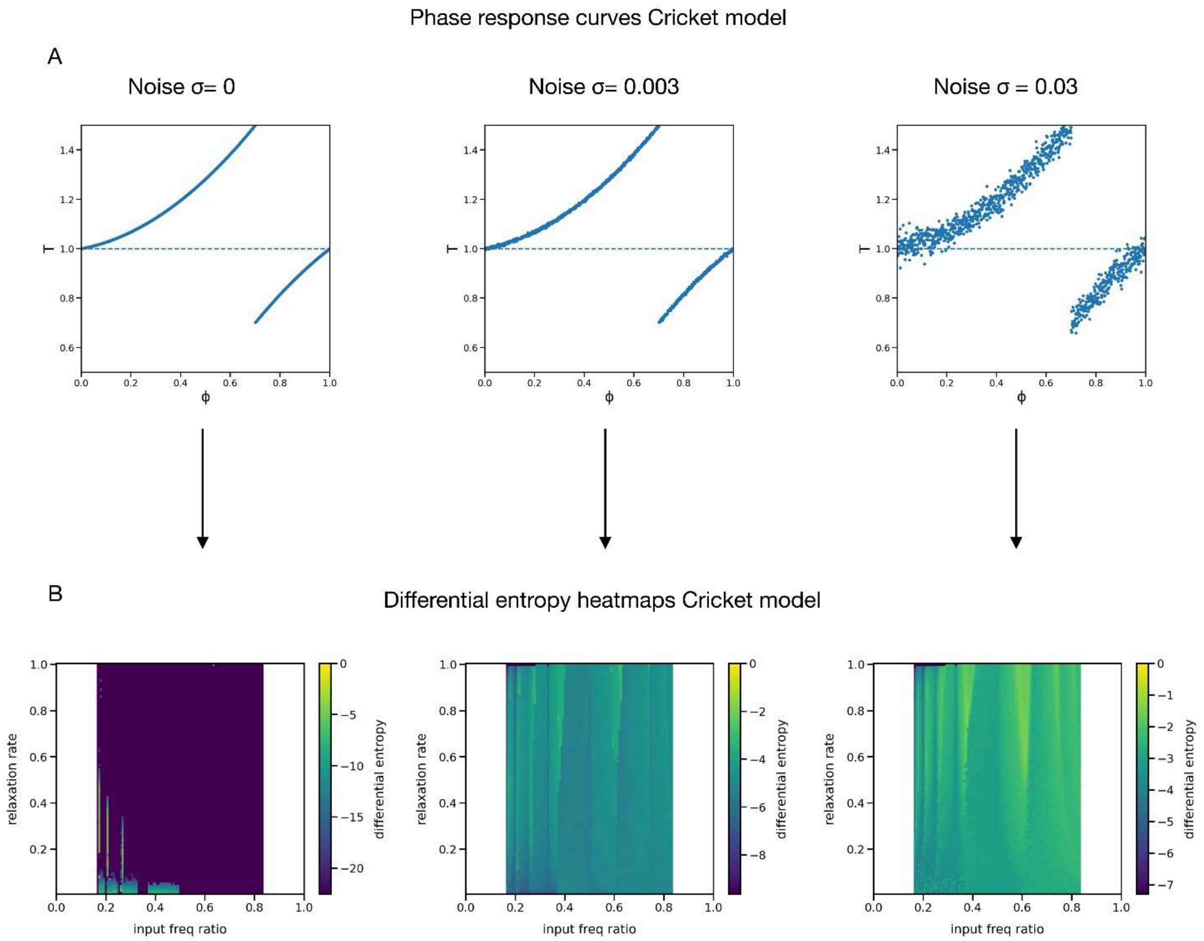

Figure S2: Detailed rhythm ratio density plots highlight the various rhythmic structures produced by the cricket model for isochronous input sequences.

A, B, C, D: Density curves of the rhythm ratios in the cricket model's output for different combinations of parameter values. Each panel represents a particular region of the parameter space as indicated with red dots or lines in the main differential entropy heatmap (analogously to Figure 4 in the main manuscript). The background shading behind each KDE curve represents the on/off-integer bins.[2] See Results S1 for a more detailed description of the results.

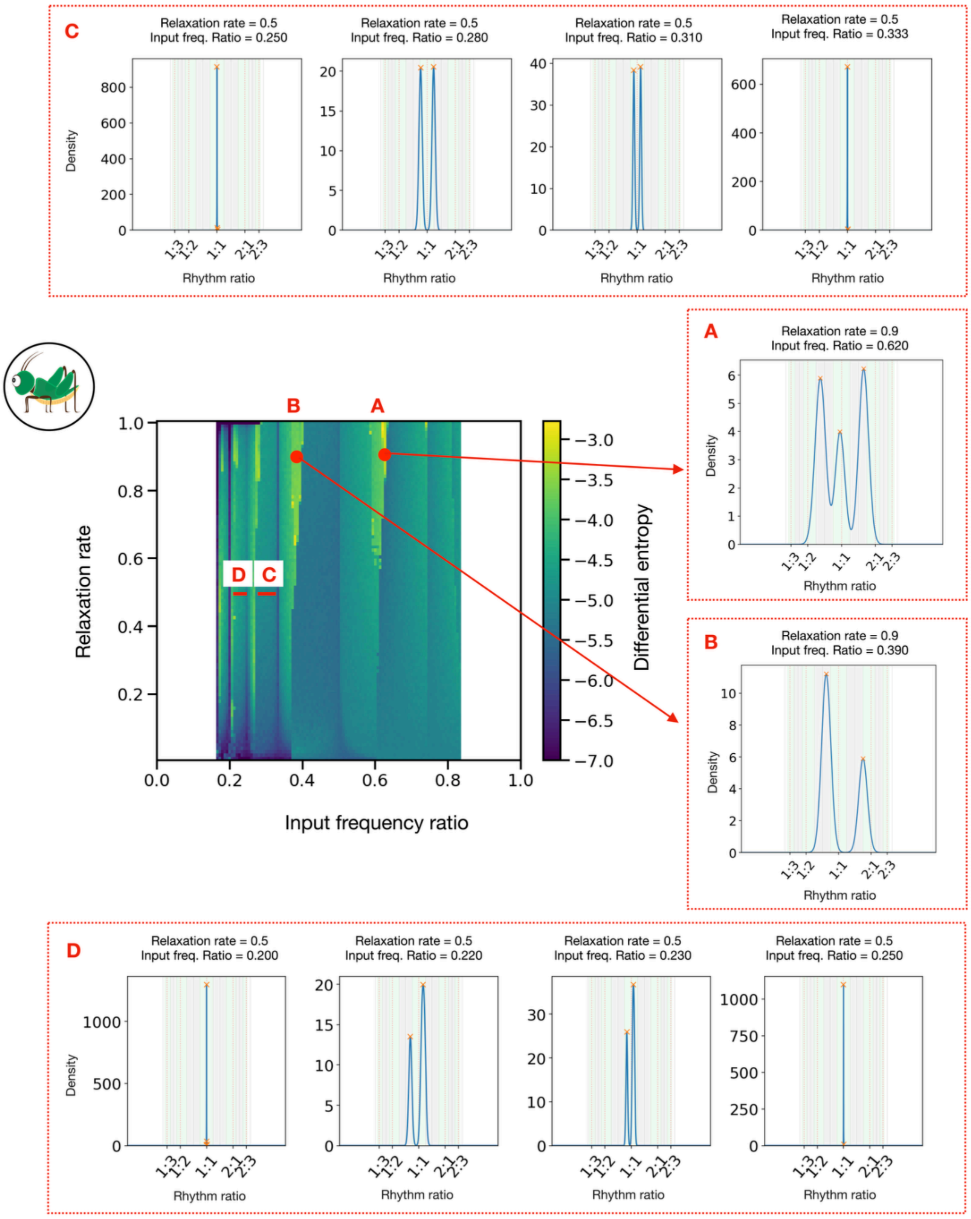

Table S1: Overview of the neuron model parameters.

These parameters, together with the state variables and equations governing them (see Table S2), define the `iaf_psc_alpha` leaky integrate-and-fire neuron model as specified in the NEST spiking neural network simulation software (version 3.8).[3] The notations and descriptions are taken from the NEST simulator documentation; for more details, see https://nest-simulator.readthedocs.io/en/v3.8/models/iaf_psc_alpha.html.

		Description
Parameters	E_L	Resting membrane potential (default value: -70 mV)

	C_m	Capacity of the membrane (default value: 250 pF)
	τ_m	Membrane time constant (default value: 10 ms)
	t_{ref}	Duration of refractory period (default value: 2 ms)
	V_{th}	Spike threshold (default value: -55 mV)
	V_{reset}	Reset potential of the membrane (default value: -70 mV)
	$\tau_{syn, ex}$	Rise time of the excitatory synaptic alpha function (default value: 2 ms)
	$\tau_{syn, in}$	Rise time of the inhibitory synaptic alpha function (default value: 2 ms)
	I_e	Constant input current (default value: 0 pA)
	V_{min}	Absolute lower value for the membrane potential (default value: $-\infty$ mV)

Table S2: Overview of the neuron model state variables and equations.

These state variables and equations, together with the model's parameters (see Table S1), define the `iaf_psc_alpha` leaky integrate-and-fire neuron model as specified in the NEST spiking neural network simulation software (version 3.8).[3] The notations and descriptions are taken from the NEST simulator documentation; for more details, see https://nest-simulator.readthedocs.io/en/v3.8/models/iaf_psc_alpha.html.

		Description
**State variables**	V_m	Membrane potential (initial value: -70 mV)
	$I_{syn,ex}$	Excitatory synaptic input current (initial value: 0 pA)
	$I_{syn,in}$	Inhibitory synaptic input current (initial value: 0 pA)
**Equations**	$\frac{dV_m}{dt} = -\frac{V_m - E_L}{\tau_m} + \frac{I_{syn} + I_e}{C_m}$	Evolution of the membrane potential V_m<br>*The change of the membrane's potential over time is influenced by the total incoming current ($I_{syn} + I_e$) charging the membrane, combined with the membrane gradually losing potential over time, proportional to the difference between its potential and the resting potential ($V_m - E_L$).*
	$V_m(t_k) < V_{th}$ and $V_m(t_{k+1}) \geq V_{th}$	Condition for emitting a spike at time step $t^* = t_{k+1}$<br>*If the membrane potential V_m increases above the threshold V_{th} between two consecutive time steps t_k and t_{k+1}, the neuron emits a spike.*
	$V_m(t) = V_{reset}$	Refractory period after spiking, $t^* \leq t < t^* + t_{ref}$<br>*After spiking, the neuron has a refractory period t_{ref} during which its membrane potential remains fixed at the reset potential V_{reset} and is not influenced by the received input current.*
	$I_{syn}(t) = I_{syn,ex}(t) + I_{syn,in}(t)$	Total post-synaptic input current<br>*The input current received from incoming spikes is the sum of the input current received from both excitatory and inhibitory spikes.*
	$I_{syn,X}(t) = \sum_j w_j \sum_k i_{syn,X}(t - t_j^k - d_j)$	Excitatory ($X = ex$) and inhibitory ($X = ex$) post-synaptic input current<br>*The total excitatory/inhibitory input current at time t equals the weighted sum of the input current from each connected neuron j. For each neuron, the share of input current is given by convolution of the response $i_{syn,X}$ with the spike train, delayed by the neuron's delay d_j.*
	$i_{syn,X}(t) = \frac{e}{\tau_{syn,X}} t e^{-(t/\tau_{syn,X})} \Theta(t)$<br>with $i_{syn,X}(t = \tau_{syn,X}) = 1$,<br>$\Theta(t) = 1$ if $t \geq 0$, and<br>$\Theta(t) = 0$ if $t < 0$	The individual post-synaptic currents (PSCs)<br>*The total amount of charge from the α-shaped synaptic input current received by a neuron is proportional to the membrane time constant parameter τ_m.*

Table S3: Overview of parameters used in computational experiment 1.
Parameter values that were varied in the experiment simulations are highlighted in grey. Non-listed neuron parameters had the default value from NEST. The non-constant parameter values varied linearly between the listed minimum and maximum in 100 steps in 100 steps, except for the neuron's constant input current. The constant input current was determined by linearly changing the ratio $f_{input} / (f_{input} + f_{model})$ from 0.1666… (1:5) to 0.8333… (5:1). The ratio between the constant input current and the neuron's intrinsic firing frequency f was determined according the following formula:

$$f = 1000\left[-\ln\left(1 - \frac{(V_{th} - V_{reset})\, C_m}{I_e - \tau_m}\right)\tau_m + t_{ref}\right]^{-1}$$

For a detailed overview of neuron parameters and equations, see Tables S1 and S2.

	Parameter	Value(s)
**Neuron model**	Constant input current (I_e)<br>*(→ Intrinsic neuron frequency)*	375 pA – 548 pA<br>*(→ 5 Hz – 74 Hz)*
	Synaptic rise time	2 ms
	Poisson generator mean firing rate	1 Hz – 370 Hz
	Excitatory connection weight	200
	Duration of simulation	10000 ms
**Cricket model**	Poisson generator mean stimulus period<br>*(relative to the intrinsic cricket period)*	0.2 – 5
	Relaxation rate	0 – 1
	Number of simulated chirps	1000

Table S4: Overview of parameters used in computational experiment 2.
Parameter values that were varied in the experiment simulations are highlighted in grey (see Tables S1, S2, and S3 for details).

	Parameter	Value(s)
**Neuron model**	Constant input current (I_e)<br>*(→ Intrinsic neuron frequency)*	375 pA – 548 pA<br>*(→ 5 Hz – 74 Hz)*
	Synaptic rise time	2 ms
	Constant input current (I_e) of input neuron<br>*(→ Intrinsic neuron frequency)*	375 pA – 5526 pA<br>(→ 5 Hz – 370 Hz)
	Excitatory connection weight	200
	Duration of simulation	10000 ms
**Cricket model**	Isochronous stimuli period<br>*(relative to the intrinsic cricket period)*	0.2 – 5
	Relaxation rate	0 – 1
	Number of simulated chirps	1000

Table S5: Overview of parameters used in computational experiment 3.
Parameter values that were varied in the experiment simulations are highlighted in grey (see Tables S1, S2, and S3 for details).

	Parameter	Value(s)
**Neuron model**	Constant input current (I_e)<br>*(→ Intrinsic neuron frequency)*	375 pA<br>*(→ 5 Hz)*
	Synaptic rise time	2ms – 8ms
	Constant input current (I_e) of input neuron<br>*(→ Intrinsic neuron frequency)*	375 pA<br>(→ 5 Hz)
	Excitatory connection weight	1 – 10000
	Duration of simulation	10000 ms

Results S1: Results of the cricket model simulations in computational experiment 2

For the cricket model, adding noise to the phase response curve (PRC; see Figure S1) prevents the model from returning to perfect isochrony after a perturbation. As such, across all parameter combinations, the resulting sequences are less organized and have a substantially higher differential entropy than the isochronous input sequence. Nevertheless, the differential entropy does vary systematically across the tested parameter space (Figure 3E). For any given relaxation rate, the differential entropy is at its lowest when the frequencies of the model and the input form an exact small-integer ratio. In these cases, the input and the cricket's intrinsic rhythm are naturally synchronized, and the cricket has a close to perfectly isochronous output (Figure 3F). Between these exact small-integer ratios, the cricket model alternates between several interval durations, repeatedly adapting its period to the input and producing a less structured outcome.

In some regions of the parameter space, for input frequency ratios between small-integer ratios, different types of rhythmic structure emerge as the model tries to synchronize with the isochronous input. In these regions, the model produces asymmetrical rhythm ratio clusters corresponding to a *short–long–very long* or *short–very short–long* interval structure (see Figure S2 B,D). In contrast, in other regions of the parameter space, the output exhibits two symmetrical rhythm ratio clusters of equal height corresponding to the alternation between two interval durations: short–long (Figure S2 C). At high relaxation rates the model reaches its highest differential entropy values. Here, the model produces similar rhythmic patterns with two or three distinct interval durations, but the rhythm ratios are less precisely clustered (Figure S2 A,B). Above the 2:1 input frequency ratio (i.e., rhythm ratio 0.66 and higher), where the frequency of the input stimuli is much higher than the cricket's rate, the model's output remains mainly isochronous across the tested input ratios.

Code fragment S1: Generative version of the cricket model [1] in Python.

```
import itertools
import numpy as np
import matplotlib.pyplot as plt


def prc(phi, discontinuity=0.7, curviness=0.75, m=0.7, M=1.5,
        noise_mu=0, noise_sigma=0.003):
   if phi <= discontinuity:
       a = curviness
       b = (M - 1) / discontinuity - a * discontinuity
       return 1 + b * phi + a * phi**2 +  np.random.normal(noise_mu, noise_sigma)
   else:
       a = -curviness
       b = (m - 1) / (1 - discontinuity) - a * (1 - discontinuity)
       return ((1 + b * (1 - phi) + a * (1 - phi)**2) +
               np.random.normal(noise_mu, noise_sigma))


def generate_isochronous_stimuli(period, start_time=1):
   for i in itertools.count():
       yield start_time + i * period


def generate_poisson_spike_train (period, start_time=1):
   time = start_time
   for i in itertools.count():
       time = time + np.random.exponential(period)
       yield time


def generate_cricket_chirp_events(stimulus_generator, nb_chirps, relaxation=1,
                                  plot=False, **kwargs):
   nb_chirps = nb_chirps + 35
   t = 0
   T = 1.0
   chirps = []
   stimuli = []
   count = 0
   next_stimulus = next(stimulus_generator, np.inf)
   while count < nb_chirps:
       if t <= next_stimulus:
           if len(chirps) > 0:
               T = relaxation + (1 - relaxation) * (t - chirps[-1])
           chirps.append(t)
           count += 1
           t += T
       else:
           prev_t = chirps[-1]
           phi = (next_stimulus - prev_t) / T
           t = prev_t + T * prc(phi, **kwargs)
           stimuli.append(next_stimulus)
           next_stimulus = next(stimulus_generator, np.inf)

   if plot:
       plt.figure(figsize=(15, 4))
       plt.xlabel('Time', fontsize=16)
       for chirp in chirps:
           plt.axvline(chirp)
       for stimulus in stimuli:
           plt.axvline(stimulus, color='r')

   chirps = np.array(chirps[35:])
   stimuli = np.array(stimuli)
   return chirps, stimuli
```

References

1. Sismondo, E. (1990). Synchronous, Alternating, and Phase-Locked Stridulation by a Tropical Katydid. *Science*, *249*(4964), 55–58.
2. Roeske, T. C., Tchernichovski, O., Poeppel, D., & Jacoby, N. (2020). Categorical Rhythms Are Shared between Songbirds and Humans. *Current Biology*, *30*(18), 3544-3555.e6. https://doi.org/10.1016/j.cub.2020.06.072
3. Graber, S., Mitchell, J., Kurth, A. C., Terhorst, D., Skaar, J.-E. W., Schöfmann, C. M., Kunkel, S., Trensch, G., Haug, N., Mallett, D., Andriyovich, P. Y., Otazu Porter, X., Lee, A. Y., & Plesser, H. E. (2024). *NEST 3.8* [Computer software]. Zenodo. https://doi.org/10.5281/zenodo.12624784
